# Crystal structure of a Tb^III^–Cu^II^ glycine­hydroxamate 15-metallacrown-5 sulfate complex

**DOI:** 10.1107/S2056989021011907

**Published:** 2021-11-18

**Authors:** Anna V. Pavlishchuk, Inna V. Vasylenko, Matthias Zeller, Anthony W. Addison

**Affiliations:** a Department of Chemistry, Taras Shevchenko National University of Kyiv, Volodymyrska str. 62, Kyiv, 01601, Ukraine; b L.V. Pisarzhevskii Institute of Physical Chemistry of the National Academy of Sciences of the Ukraine, Prospect Nauki 31, Kiev 03028, Ukraine; c Department of Chemistry, Purdue University, 560 Oval Drive, West Lafayette, IA 47907-2084, USA; d Department of Chemistry, Drexel University, Philadelphia, PA 19104-2816, USA

**Keywords:** crystal structure, terbium(III), copper(II), metallamacrocycle, 15-metallacrown-5

## Abstract

The core of the title complex, bis­[hexa­aqua­hemi­aqua­penta­kis­(μ_3_-glycine­hydroxamato)sulfato­penta­copper(II)terbium(III)] sulfate hexa­hydrate, [TbCu_5_(SO_4_)(GlyHA)_5_(H_2_O)_6.5_]_2_(SO_4_)·6H_2_O (**1**), which belongs to the 15-metalla­crown-5 family, consists of five glycine­hydroxamate dianions (GlyHA^2−^; C_2_H_4_N_2_O_2_) and five copper(II) ions linked together forming a metallamacrocyclic moiety. The terbium(III) ion is connected to the centre of the metallamacrocycle through five hydroxamate oxygen atoms. The coordination environment of the Tb^3+^ ion is completed to an octa­coordination level by oxygen atoms of a bidentate sulfate and an apically coordinated water mol­ecule, while the copper(II) atoms are square-planar, penta- or hexa­coordinate due to the apical coordination of water mol­ecules. Continuous shape calculations indicate that the coordination polyhedron of the Tb^3+^ ion in **1** is best described as square anti­prismatic. The positive charge of each pair of [TbCu_5_(GlyHA)_5_(H_2_O)_6.5_(SO_4_)]_2_^2+^ fragments is compensated by a non-coordinated sulfate anion, which is located on an inversion center with 1:1 disordered oxygen atoms. Complex **1** is isomorphous with the previously reported compounds [*Ln*Cu_5_(GlyHA)_5_(SO_4_)(H_2_O)_6.5_]_2_(SO_4_), where *Ln* ^III^ = Pr, Nd, Sm, Eu, Gd, Dy and Ho.

## Abstract 2

The metallamacrocyclic core of the discrete hexa­nuclear 15-metallacrown-5 complex [TbCu_5_(GlyHA)_5_(H_2_O)_6.5_(SO_4_)]_2_(SO_4_)·6H_2_O contains five copper(II) ions linked by five glycine­hydroxamate (GlyHA^2–^) dianions with a square-anti­prismatically octa­coordinate terbium(III) ion in the centre. The positive charge of the 15-metallacrown-5 [TbCu_5_(GlyHA)_5_]^3+^ core is compensated by bidentate and non-coordinated sulfate anions.

## Chemical context

Numerous research studies devoted to polynuclear *3d*–4*f* assemblies have been stimulated by their non-trivial lumin­escence properties (Jankolovits *et al.*, 2011 ▸; Maity *et al.*, 2015 ▸), single-mol­ecule magnet (SMM) behaviour (Dhers *et al.*, 2016 ▸; Zangana *et al.*, 2014 ▸) and their significant magnetocaloric effect (Pavlishchuk & Pavlishchuk, 2020 ▸; Zheng *et al.*, 2014 ▸). The 15-metallacrown-5 complexes are *3d*–4*f* metallamacrocyclic assemblies, which can be easily obtained from one-step reactions between an *α*-substituted hydroxamic acid and the corresponding salts of transition metals and lanthanides (Stemmler *et al.*, 1999 ▸; Pavlishchuk *et al.*, 2011 ▸, 2019 ▸). Compounds bearing 15-metallacrown-5 {*Ln*Cu_5_}^3+^ units have demonstrated the ability to serve as sensors (Zabrodina *et al.*, 2018 ▸), can absorb and adsorb various small mol­ecules (Lim *et al.*, 2010 ▸; Pavlishchuk *et al.*, 2014 ▸; Ostrowska *et al.*, 2016 ▸) and display SMM behaviour (Wang *et al.*, 2019 ▸, 2021 ▸; Zaleski *et al.*, 2006 ▸; Wu *et al.*, 2021 ▸). Taking into account the fact that 15-metallacrowns-5 are also suitable building blocks for the generation of porous coordination polymers and discrete assemblies (Pavlishchuk *et al.*, 2017*a* ▸,*b* ▸, 2018 ▸), the synthesis of new examples of this class of metallamacrocyclic assemblies and studies of their structural features are of particular inter­est. Herein we report the crystal structure of the new 15-metallacrown-5 complex [TbCu_5_(GlyHA)_5_(H_2_O)_6.5_(SO_4_)]_2_ (SO_4_)·13(H_2_O) (**1**), which complements the previously reported series of isomorphous metallamacrocycles with Pr, Nd, Sm, Eu, Gd, Dy and Ho ions at their centres. 
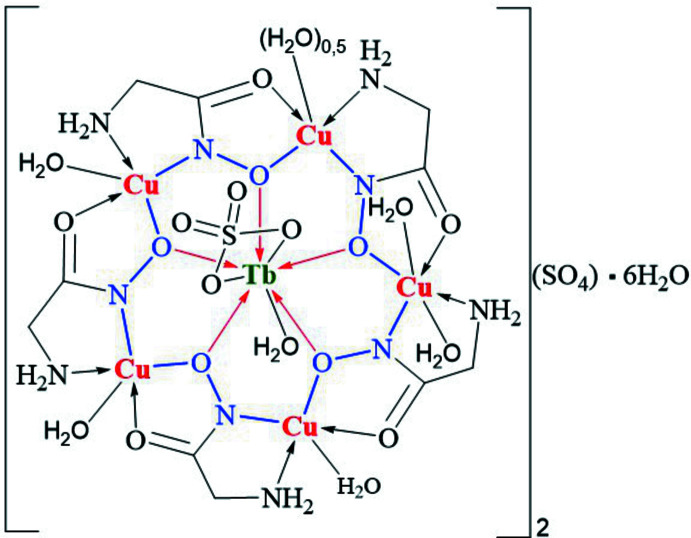


## Structural commentary

**Figure 1:**
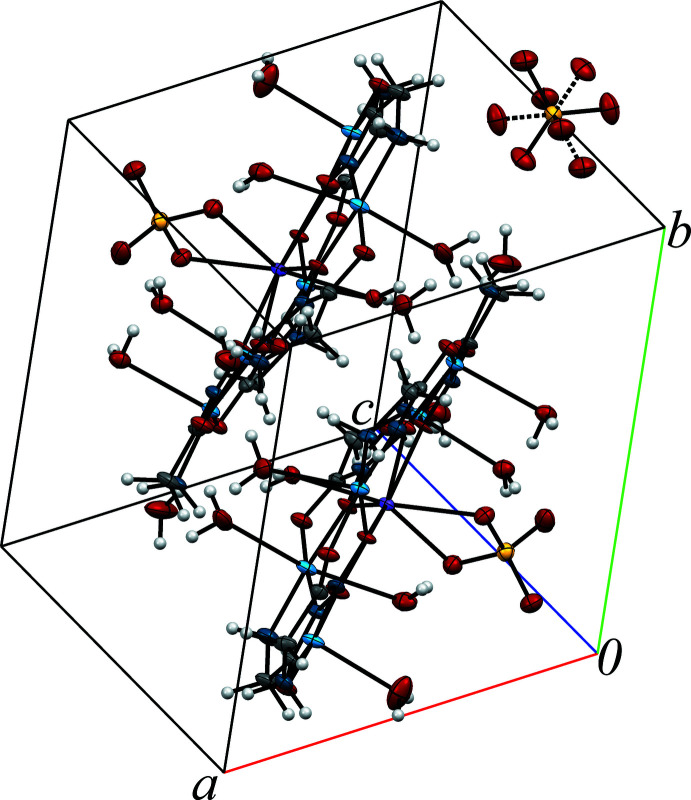
The unit cell of complex **1** containing two [TbCu_5_(GlyHA)_5_(SO_4_)(H_2_O)_6.5_]^+^ metallacrown cations and non-coordinated sulfate anions (located on a inversion center with O atoms 1:1 disordered). Non-coordinated water mol­ecules are omitted for clarity of presentation.

Complex **1** crystallizes in the space group *P* 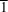 and is isostructural with the previously reported complexes [*Ln*Cu_5_(GlyHA)_5_(SO_4_)(H_2_O)_6.5_]_2_(SO_4_), where GlyHA^2−^ is the dianion of glycine­hydroxamic acid and *Ln* ^III^ = Pr, Nd, Sm, Eu, Gd, Dy and Ho (Pavlishchuk *et al.*, 2011 ▸). Each unit cell in **1** contains two [TbCu_5_(GlyHA)_5_(SO_4_)(H_2_O)_6.5_]^+^ 15-metallacrown-5 cations related by an inversion center, one non-coordinated sulfate anion for charge-balance and non-coord­inated water mol­ecules (Figs. 1 ▸ and 2 ▸).

The core of the [TbCu_5_(GlyHA)_5_(SO_4_)(H_2_O)_6.5_]^+^ complex cation in **1** is constructed from five copper(II) ions linked by five bridging glycine­hydroxamate dianions (GlyHA^2−^) and a terbium(III) ion bound at the centre of the metallocycle (Fig. 1 ▸). The copper(II) equatorial coordination environment in **1** is formed by two oxygen atoms (from a carboxyl­ate and a deprotonated hydroxamate group) and two nitro­gen atoms (from an amine and a deprotonated hydroxamate). The equatorial Cu—O_eq_ and Cu—N_eq_ distances range from 1.928 (3) to 1.969 (3) Å and 1.890 (4) to 2.018 (4) Å (Table 1 ▸), respectively, which is typical of amino­hydroxamate 15-metallacrown-5 complexes (Stemmler *et al.*, 1999 ▸; Pavlishchuk *et al.*, 2011 ▸; Katkova *et al.*, 2015*a* ▸; Meng *et al.*, 2016 ▸). As a result of the apical coordination of water mol­ecules to copper(II) ions, Cu1 has distorted square-bipyramidal coordination [Cu1—O20 = 2.601 (4) Å and Cu1—O21 = 2.736 (4) Å], while Cu3, Cu4 and Cu5 are in square-pyramidal environments [Cu3—O16 = 2.508 (4) Å, Cu4—O17 = 2.481 (4) Å and Cu5—O18 = 2.379 (4) with *τ*-values (Addison *et al.*, 1984 ▸) ranging from 0.07 to 0.13]. As a result of the disorder of the O19 water mol­ecule between two symmetry-equivalent positions with occupancy factors of 0.5, 50% of the Cu2 atoms in **1** have square-planar coordination environments, while the other 50% possess a square-pyramidal coordination [Cu2—O19 = 2.409 (10), *τ* = 0.022 (Addison *et al.*, 1984 ▸)]. The terbium(III) ions at the centres of the [Cu_5_(GlyHA)_5_] metallamacrocyclic cores in **1** are bound by five hydroxamate oxygen atoms. The Tb—O_eq_ bond lengths are typical for 15-metallacrown-5 complexes and range from 2.370 (3) to 2.430 (3) Å (Stemmler *et al.*, 1999 ▸; Pavlishchuk *et al.*, 2011 ▸; Katkova *et al.*, 2015*a* ▸; Meng *et al.*, 2016 ▸).

**Figure 3:**
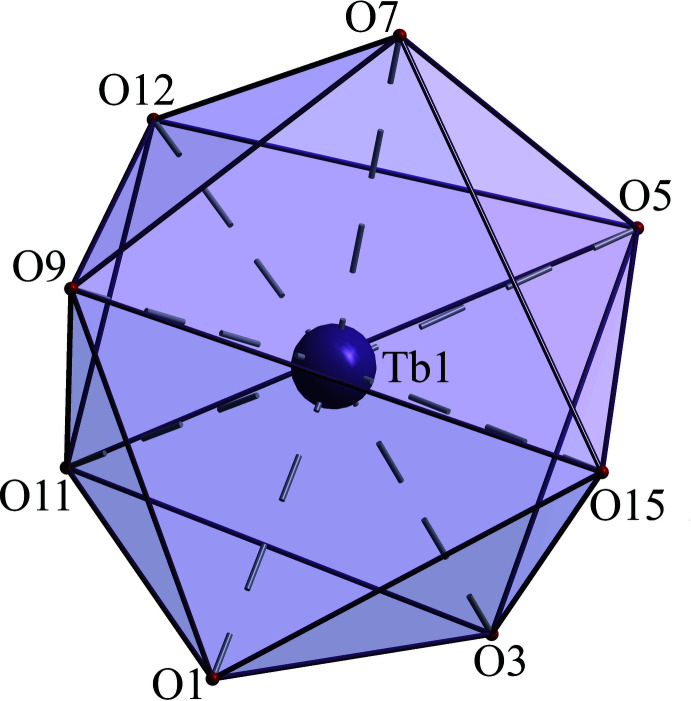
The Tb^III^ coordination sphere geometry in **1**.

The coordination environment of the Tb^3+^ ion is completed to an octa­coordination level *via* the two oxygen atoms O11 [Tb1—O11 = 2.451 (3) Å] and O12 [Tb1—O12 = 2.436 (3) Å] from the bidentate sulfate anions and O15 [Tb1—O15 = 2.383 (3) Å] from a water mol­ecule coordinated in the *trans*-position opposite to the SO_4_^2−^ ion. An analysis of selected structural parameters for complex **1** and those of isomorphous compounds with other *Ln* ^III^ ions (Table 2 ▸) reveals the influence of the lanthanide contraction. Similar behaviour was found in other series of lanthanide(III) containing metallamacrocycles (Pavlishchuk *et al.*, 2011 ▸; Zaleski *et al.*, 2011 ▸). According to *Shape 2.1* (Casanova *et al.*, 2005 ▸) calculations (Fig. 3 ▸, Table 3 ▸), the coordination geometry of the Tb^III^ ion in **1** is a square anti­prism (*D*_4*d*_), which is of particular inter­est with respect to potential generation of lanthanide(III)-containing SMMs (Liu *et al.*, 2018 ▸). The deviations from an idealized square-anti­prismatic geometry in the [*Ln*Cu_5_(GlyHA)_5_(SO_4_)(H_2_O)_6.5_]_2_(SO_4_) complexes decrease with reduction of the deviation of the *Ln* ^III^ ion from the mean plane of the metallacrown core, which parallels the ionic radii of the *Ln* ^III^ ions (Table 3 ▸). It may be noted that, in the case of a series of related 15-metallacrown-5 complexes with octa­coordinate *Ln* ^III^ ions containing bidentate carbonates or acetates instead of sulfates, the coordination of the lanthanide ions is triangular dodeca­hedral (*D*_2*d*_) (Table 3 ▸).

The Cu⋯Cu and Ln⋯Cu separations for complex **1** range from 4.501 (1) to 4.577 (1) Å and 3.8398 (8) to 3.8944 (8) Å, respectively, and are typical for {*Ln*Cu_5_}^3+^ metallacrowns (Stemmler *et al.*, 1999 ▸; Pavlishchuk *et al.*, 2011 ▸; Katkova *et al.*, 2015*a* ▸; Meng *et al.*, 2016 ▸). The Cu—O, Cu—N and Cu⋯Cu distances do not vary significantly amongst metallamacrocycles with different bidentate counter-anions (Table 2 ▸). The metallacrown moiety in **1** is close to planar, the deviation of Tb^III^ ions from the mean plane Cu1–Cu5 being 0.4270 (4) Å. The *Ln*—O distances, *Ln*—Cu separations and deviations of the *Ln* ^III^ ions from the Cu_5_ planes of the metallamacrocycles trend with the lanthanide contraction in all members of the isomorphous [*Ln*Cu_5_(GlyHA)_5_]^3+^ series. However, there are some minor differences in the observed values for a given *Ln* ^III^ ion, depending on the coordinated bidentate counter-anion, which is likely associated with the different planarities of the {*Ln*Cu_5_}^3+^ cores (Table 2 ▸).

## Supra­molecular features

The [LnCu_5_(GlyHA)_5_]^3+^ cations in complex **1** are non-oligomerized, which is typical for 15-metallacrown-5 complexes. The water apical to Tb^III^ in **1** (O15) is involved in the formation of intra­molecular hydrogen bonds (O15—H15*A*⋯O21 and O15—H15*B*⋯O16) with apically coordinated water mol­ecules O16 and O21 on copper(II) ions Cu3 and Cu1, respectively. Intra­molecular hydrogen bonds in **1** are also formed between the bidentate sulfate and apically coord­inated water mol­ecules O17, O18 and O20 (O17—H17*A*⋯O12, O18—H18*B*⋯O14 and O20—H20*B*⋯O11) on copper(II) ions Cu4, Cu5 and Cu1. An extended system of inter­molecular hydrogen bonds [N2—H2*A*⋯O15^iii^, N8—H8*B*⋯O12^vi^ (SO_4_), N10—H10*A*⋯O20^i^, O10^iiii^⋯H21*B*—O21, O6^vi^⋯H17*B*—O17, O21—H21*A*⋯O18^iv^, O16—H16*A*⋯O17^iv^] links adjacent [TbCu_5_(GlyHA)_5_(H_2_O)_6.5_(SO_4_)]^+^ cations and non-coordinated sulfate anions [N4—H4*A*⋯O27^iv^(SO_4_), O18—H18*A*⋯O27(SO_4_), N4—H4*A*⋯O25^x^(SO_4_) and O20—H20*A*⋯O25(SO_4_)]. Non-coordinated water mol­ecules in **1** are linked by hydrogen bonds with carbonyl oxygen and amine nitro­gen atoms in the glycine­hydroxamate unit from the metallacrown core (O4^*i*^⋯H23*A*—O23, O8⋯H24*B*—O24, N6—H6*B*⋯O24^vi^, N8—H8*A*⋯O23, N10—H10*B*⋯O22^viii^), apically coordinated water mol­ecules (O16—H16*B*⋯O22, O19—H19*A*⋯O24^*vii*^, O19–*-*H19*B*⋯O24^*vi*^) or bidentate sulfate (O11^i^⋯H24*A-*–O24 and O13^ii^⋯H23*B*—O23). Hydrogen-bond parameters and symmetry codes are given in Table 4 ▸.

## Database survey

Compounds most closely related to **1** are its isomorphous counterparts [*Ln*Cu_5_(GlyHA)_5_(SO_4_)(H_2_O)_6.5_]_2_(SO_4_), where GlyHA^2−^ is the dianion of glycine­hydroxamic acid and *Ln* ^III^ = Pr, Nd, Sm, Eu, Gd, Dy and Ho (Pavlishchuk *et al.*, 2011 ▸). A search of the Cambridge Structural Database (Version 5.41, 2021; Groom *et al.*, 2016 ▸) reveals other compounds that also feature an *Ln*Cu_5_(GlyHA)_5_ core, with counter-anions such as nitrate, acetate, chloride, lactate, carbonate, sulfate, isophthalate, terephthalate and all lanthanide ions other than radioactive Pm (Katkova *et al.*, 2015*a* ▸,*b* ▸; Pavlishchuk *et al.*, 2011 ▸, 2017*a* ▸, Pavlishchuk *et al.*, 2018 ▸, 2019 ▸; Stemmler *et al.*, 1999 ▸; Muravyeva *et al.*, 2016 ▸; Kremlev *et al.*, 2016 ▸). Most of these complexes feature, similar to **1**, individual mol­ecular complex cations (Katkova *et al.*, 2015*a* ▸,*b* ▸; Pavlishchuk *et al.*, 2011 ▸, 2017*a* ▸, 2018 ▸, 2019 ▸; Stemmler *et al.*, 1999 ▸; Muravyeva *et al.*, 2016 ▸; Kremlev *et al.*, 2016 ▸), but a small number of oligomerized examples have also been reported (Pavlishchuk *et al.*, 2017*a* ▸, 2018 ▸).

## Synthesis and crystallization

Complex **1** was synthesized and crystallized according a general procedure described previously (Pavlishchuk *et al.*, 2011 ▸). Single crystals were obtained by slow evaporation from an aqueous solution of **1**.

## Refinement

Crystal data, data collection and structure refinement details are summarized in Table 5 ▸. The structure is isomorphous with its Dy, Eu, Gd, Ho, Nd, Pr analogues (Pavlishchuk *et al.*, 2011 ▸) and was solved by isomorphous replacement. The O19 water mol­ecule is disordered over two mutually exclusive positions across an inversion center and was refined as half occupied. The non-coordinated sulfate ion is located on an inversion center and the oxygen atoms are disordered over two sets of positions with half occupancy.

C—H bond distances were constrained to 0.99 for aliphatic CH_2_ moieties. N—H bond distances were constrained to 0.91 Å for pyramidal (*sp* ^3^-hybridized) ammonium NH_2_^+^ groups. Water H-atom positions were refined, and O—H distances were restrained to 0.84 (2) Å. The H⋯H distances within the O23 and O24 water mol­ecules were further restrained to 1.35 (2) Å. *U*_iso_(H) values were set to *kU*_eq_(C/N/O) with *k* =1.5 for OH, and 1.2 for CH_2_ and NH_2_^+^ units, respectively.

## Supplementary Material

Crystal structure: contains datablock(s) I, global. DOI: 10.1107/S2056989021011907/yy2004sup1.cif

Structure factors: contains datablock(s) I. DOI: 10.1107/S2056989021011907/yy2004Isup2.hkl

CCDC reference: 2121203

Additional supporting information:  crystallographic information; 3D view; checkCIF report

## Figures and Tables

**Figure 2:**
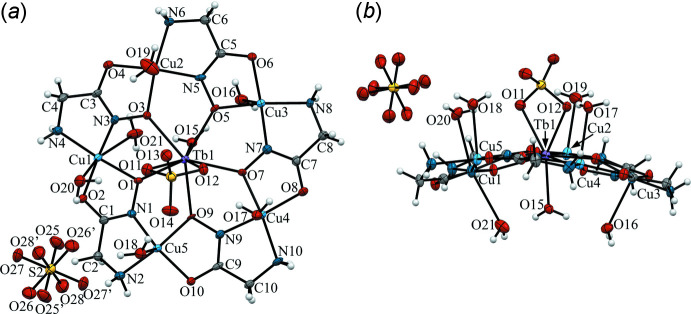
Structure of the [TbCu_5_(GlyHA)_5_(SO_4_)(H_2_O)_6.5_]^+^ metallacrown cations in **1**. The dashed lines indicate the disorder of the non-coordinated sulfate anion. Displacement ellipsoids are shown at the 50% probability level. [Symmetry code: (i) *x*, *y*, *z* + 1.]

**Table 1:** Selected bond lengths (Å)

Cu1—N3	1.915 (4)	Cu4—O8	1.940 (3)
Cu1—O1	1.928 (3)	Cu4—O7	1.947 (3)
Cu1—O2	1.969 (3)	Cu4—N10	2.012 (4)
Cu1—N4	1.991 (4)	Cu4—O17	2.481 (4)
Cu1—O20	2.601 (4)	Cu5—N1	1.890 (4)
Cu1—O21	2.736 (4)	Cu5—O9	1.943 (3)
Cu2—N5	1.900 (4)	Cu5—O10	1.946 (3)
Cu2—O3	1.928 (3)	Cu5—N2	2.003 (4)
Cu2—O4	1.936 (3)	Cu5—O18	2.379 (4)
Cu2—N6	2.018 (4)	Tb1—O9	2.370 (3)
Cu2—O19	2.409 (10)	Tb1—O1	2.372 (3)
Cu3—N7	1.904 (4)	Tb1—O15	2.383 (3)
Cu3—O6	1.944 (3)	Tb1—O3	2.386 (3)
Cu3—O5	1.949 (3)	Tb1—O7	2.411 (3)
Cu3—N8	2.014 (4)	Tb1—O5	2.430 (3)
Cu3—O16	2.508 (4)	Tb1—O12	2.436 (3)
Cu4—N9	1.894 (4)	Tb1—O11	2.451 (3)

**Table 2:** Comparison of the structural characteristics (Å, °) of {*Ln*Cu_5_}^3+^ 15-metallacrown-5 complexes with octa­coordinate *Ln* ^III^ ions and various bidentate anions

Complex*^*a*^*	Cu—O/N_eq_	*Ln*—O_eq_	*Ln*—O_aq_	*Ln*⋯Cu	Cu⋯Cu	Deviation of *Ln* ^III^ from Cu_5_ plane	*Ln*O_8_ geometry*^*b*^*
Pr—SO_4_	1.898 (2)–2.013 (2)	2.4247 (18)–2.4716 (18)	2.495 (2)–2.528 (2)	3.862 (3)–3.923 (2)	4.530 (2)–4.604 (2)	0.459	SAPR-8
Nd—SO_4_	1.898 (2)–2.0156 (19)	2.4145 (16)–2.4642 (16)	2.4787 (18)–2.5108 (17)	3.862 (3)–3.915 (4)	4.524 (4)–4.598 (5)	0.452	SAPR-8
Sm—SO_4_	1.900 (4)–2.015 (4)	2.398 (3) −2.450 (3)	2.441 (4)–2.484 (4)	3.8539 (9)–3.9083 (8)	4.518 (1)–4.592 (1)	0.439	SAPR-8
Eu—SO_4_	1.896 (3)–2.013 (3)	2.389 (3)–2.437 (3)	2.431 (3)–2.467 (3)	3.844 (7)–3.899 (8)	4.504 (8)–4.585 (9)	0.439	SAPR-8
Eu—CO_3_	1.886 (14)–2.022 (13)	2.406 (11)–2.493 (11)	2.369 (13)–2.392 (15)	3.890 (2)–3.911 (3)	4.575 (3)–4.589 (3)	0.351	TDD-8
Eu-OAc	1.902 (3)–2.041 (2)	2.440 (4)–2.515 (2)	2.4057 (18)–2.443 (2)	3.8517 (4)–3.9049 (4)	4.5664 (5)–4.6074 (4)	0.469	TDD-8
Gd—SO_4_	1.892 (3)–2.014 (3)	2.378 (3)–2.434 (3)	2.398 (3)–2.452 (3)	3.838 (7)–3.897 (9)	4.501 (8)–4.578 (11)	0.430	SAPR-8
Gd—CO_3_	1.898 (2)–2.022 (2)	2.381 (2)–2.484 (2)	2.288 (17)–2.396 (10)	3.8699 (5)–3.9097 (5)	4.5677 (7)–4.5846 (7)	0.337	TDD-8
Gd-OAc	1.890 (12)–2.041 (11)	2.393 (3)–2.438 (9)	2.426 (10)–2.512 (10)	3.845 (2)–3.897 (2)	4.562 (2)–4.602 (2)	0.458	TDD-8
Tb—SO_4_	1.890 (4)–2.018 (4)	2.370 (3)–2.430 (3)	2.383 (3)–2.451 (3)	3.8398 (8)–3.8944 (8)	4.501 (1)–4.577 (1)	0.427	SAPR-8
Tb-OAc	1.889 (11)–2.036 (11)	2.383 (9)–2.431 (9)	2.409 (10)–2.488 (10)	3.840 (2)–3.896 (2)	4.562 (2)–4.598 (2)	0.445	TDD-8
Dy—SO_4_	1.8908 (18)–2.0206 (19)	2.3640 (15) −2.4234 (15)	2.3665 (17)–2.4334 (17)	3.834 (2)–3.889 (2)	4.493 (2)–4.573 (2)	0.424	SAPR-8
Dy—CO_3_	1.898 (3)–2.022 (3)	2.382 (3)–2.469 (3)	2.27 (2)–2.380 (8)	3.8715 (5)–3.9016 (6)	4.5645 (7)–4.5797 (8)	0.354	TDD-8
Ho—SO_4_	1.887 (3)–2.016 (3)	2.356 (2)–2.416 (2)	2.357 (2)–2.417 (2)	3.827 (2)–3.884 (2)	4.485 (2)–4.565 (2)	0.422	SAPR-8
Ho—CO_3_	1.898 (2)–2.022 (2)	2.374 (2)–2.475 (2)	2.30 (3)–2.374 (12)	3.8670 (5)–3.9021 (5)	4.5583 (7)–4.5808 (7)	0.330	TDD-8

Notes: (*a*) Complex Tb—SO_4_ is **1**; *Ln*-SO_4_ correspond to the [*Ln*Cu_5_(GlyHA)_5_(SO_4_)(H_2_O)_6.5_]_2_(SO_4_) series with *Ln* = Pr, Nd, Sm, Eu, Gd, Dy and Ho described in (Pavlishchuk *et al.*, 2011 ▸); *Ln*-CO_3_ are [*Ln*Cu_5_(GlyHA)_5_(CO_3_)(NO_3_)(H_2_O)_5_] with *Ln* = Eu, Gd, Dy and Ho described in Pavlishchuk *et al.* (2019 ▸) and Stemmler *et al.* (1999 ▸); *Ln*-OAc are [*Ln*Cu_5_(GlyHA)_5_(OAc)(H_2_O)_5_](NO_3_)_2_ *Ln* = Eu, Gd and Tb described in Katkova *et al.* (2015*a* ▸) and Meng *et al.* (2016 ▸). (*b*) *Ln*O_8_ geometries: SAPR-8 = square anti­prism (*D*4*d*) and TDD-8 = triangular dodeca­hedron (*D*_2*d*_).

**Table 3:** Continuous shape calculations for octa­coordinated *Ln* ^3+^ ions in **1** obtained with *Shape 2.1* software (Casanova *et al.*, 2005 ▸)

	OP-8	HPY-8	HBPY-8	CU-8	SAPR-8	TDD-8	JGBF-8	JETBPY-8
Pr–SO_4_	30.846	22.755	15.952	11.561	2.215	2.397	13.029	25.482
Nd–SO_4_	30.677	22.888	15.968	11.587	2.141	2.364	13.033	25.516
Sm–SO_4_	30.387	22.903	15.951	11.562	2.020	2.311	13.013	25.752
Eu–SO_4_	30.516	23.164	16.270	11.783	1.952	2.363	13.190	25.864
Gd–SO_4_	30.465	23.110	16.032	11.570	1.907	2.269	13.151	26.121
Tb–SO_4_	30.381	23.117	16.159	11.666	1.854	2.322	13.140	26.276
Dy–SO_4_	30.357	23.195	16.112	11.603	1.799	2.254	13.168	26.433
Ho–SO_4_	30.272	23.212	16.095	11.588	1.761	2.247	13.186	26.496

Octa­coordinated ions: OP-8 = octa­gon (*D*_8*h*_); HPY-8 = hepta­gonal pyramid (*C*_7*v*_); HBPY-8 = hexa­gonal bipyramid (*D*_6*h*_); CU-8 = cube (*O*_*h*_); SAPR-8 = square anti­prism (*D*_4*d*_); TDD-8 = triangular dodeca­hedron (*D*_2*d*_); JGBF-8 = Johnson gyrobifastigium J26 (*D*_2*d*_); JETBPY-8 = Johnson elongated triangular bipyramid J14 (*D*_3*h*_).

**Table 4:** Hydrogen-bond geometry (Å, °)

*D*—H⋯*A*	*D*—H	H⋯*A*	*D*⋯*A*	*D*—H⋯*A*
O24—H24*B*⋯O8	0.84 (2)	2.01 (3)	2.807 (5)	159 (7)
O24—H24*A*⋯O11^i^	0.84 (2)	2.21 (3)	3.015 (5)	162 (7)
O23—H23*B*⋯O13^ii^	0.85 (2)	2.02 (3)	2.853 (5)	166 (6)
O23—H23*A*⋯O4^i^	0.84 (2)	1.89 (2)	2.734 (5)	176 (7)
O22—H22*B*⋯O23	0.84 (2)	1.89 (3)	2.701 (6)	162 (8)
O22—H22*A*⋯O26^iii^	0.84 (2)	2.18 (4)	2.968 (9)	155 (8)
O22—H22*A*⋯O28^ii^	0.84 (2)	1.92 (3)	2.733 (9)	161 (8)
O21—H21*B*⋯O10^iii^	0.83 (2)	1.91 (3)	2.728 (5)	165 (8)
O21—H21*A*⋯O18^iv^	0.84 (2)	1.94 (3)	2.765 (5)	167 (7)
O20—H20*B*⋯O11	0.83 (2)	2.14 (3)	2.960 (5)	168 (7)
O20—H20*A*⋯O26^v^	0.83 (2)	2.09 (3)	2.916 (9)	170 (7)
O20—H20*A*⋯O25	0.83 (2)	2.02 (5)	2.719 (9)	142 (7)
O19—H19*B*⋯O24^vi^	0.84 (2)	2.07 (9)	2.866 (11)	157 (22)
O19—H19*A*⋯O24^vii^	0.84 (2)	1.72 (7)	2.535 (12)	162 (21)
O18—H18*B*⋯O14	0.83 (2)	1.90 (2)	2.732 (5)	173 (7)
O18—H18*A*⋯O26^v^	0.84 (2)	2.04 (3)	2.857 (9)	163 (7)
O18—H18*A*⋯O27	0.84 (2)	1.91 (4)	2.648 (9)	146 (6)
O17—H17*B*⋯O6^vi^	0.83 (2)	1.90 (2)	2.730 (5)	176 (7)
O17—H17*A*⋯O12	0.83 (2)	2.10 (3)	2.905 (5)	163 (6)
O16—H16*B*⋯O22	0.84 (2)	1.89 (2)	2.721 (6)	173 (7)
O16—H16*A*⋯O17^iv^	0.84 (2)	1.95 (2)	2.784 (5)	172 (7)
O15—H15*B*⋯O16	0.84 (2)	1.86 (2)	2.692 (5)	170 (6)
O15—H15*A*⋯O21	0.84 (2)	1.85 (3)	2.668 (5)	166 (6)
N10—H10*B*⋯O22^viii^	0.91	2.13	2.920 (6)	145
N10—H10*A*⋯O20^i^	0.91	2.24	2.987 (5)	139
N8—H8*B*⋯O12^vi^	0.91	2.04	2.937 (5)	168
N8—H8*A*⋯O23	0.91	2.20	3.031 (5)	152
N6—H6*B*⋯O13^ix^	0.91	2.64	3.363 (5)	137
N6—H6*B*⋯O24^vi^	0.91	2.24	2.984 (6)	139
N6—H6*A*⋯O13^iv^	0.91	2.25	3.158 (5)	175
N4—H4*B*⋯O2^x^	0.91	2.33	3.182 (5)	156
N4—H4*A*⋯O27^iv^	0.91	2.18	3.037 (9)	156
N4—H4*A*⋯O25^x^	0.91	2.01	2.789 (9)	143
N2—H2*B*⋯O27	0.91	2.55	3.418 (9)	159
N2—H2*B*⋯O28^v^	0.91	2.08	2.868 (9)	144
N2—H2*A*⋯O15^iii^	0.91	2.07	2.946 (5)	162

Symmetry codes: (i) x, y+1, z; (ii) x+1, y+1, z; (iii) -x+1, -y+1, -z+1; (iv) x+1, y, z; (v) -x, -y, -z+1; (vi) -x+1, -y+1, -z; (vii) x, y-1, z; (viii) x-1, y, z; (ix) -x+1, -y, -z; (x) -x+1, -y, -z+1.

**Table 5:** Experimental details

Crystal data	
Chemical formula	[TbCu_5_(C_2_H_4_N_2_O_2_)_5_(SO_4_)(H_2_O)_6.5_]_2_(SO_4_)·6H_2_O
*M*_r_	2464.44
Crystal system, space group	Triclinic, *P*\overline{1}
Temperature (K)	150
*a*, *b*, *c* (Å)	9.6370 (4), 11.5888 (5), 16.2367 (6)
α, β, γ (°)	99.6716 (13), 91.3031 (12), 105.3123 (12)
*V* (Å^3^)	1719.80 (12)
*Z*	1
Radiation type	Cu *K*α
μ (mm^−1^)	15.11
Crystal size (mm)	0.20 × 0.20 × 0.08

Data collection	
Diffractometer	Bruker AXS D8 Quest CMOS diffractometer with PhotonII charge-integrating pixel array detector (CPAD)
Absorption correction	Multi-scan (*SADABS*; Krause *et al.*, 2015 ▸
*T*_min_, *T*_max_	0.454, 0.754
No. of measured, independent and observed [*I* > 2σ(*I*)] reflections	16278, 7029, 6786
*R*_int_	0.050
(sin θ/λ)_max_ (Å^−1^)	0.639

Refinement	
*R*[*F* ^2^ > 2σ(*F* ^2^)], *wR*(*F* ^2^), *S*	0.041, 0.118, 1.10
No. of reflections	7029
No. of parameters	562
No. of restraints	22
H-atom treatment	H atoms treated by a mixture of independent and constrained refinement
Δρ_max_, Δρ_min_ (e Å^−3^)	1.59, −1.34

Computer programs: *APEX3* and *SAINT* (Bruker, 2018 ▸), *SHELXS97* (Sheldrick, 2008 ▸), *SHELXL2018/3* (Sheldrick, 2015 ▸), *shelXle* (Hübschle *et al.*, 2011 ▸) and *publCIF* (Westrip, 2010 ▸).

## supplementary crystallographic information

### Crystal data

**Table:**

[TbCu_5_(C_2_H_4_N_2_O_2_)_5_(SO_4_)(H_2_O)_6.5_]_2_(SO_4_)·6H_2_O	*Z* = 1
*M**_r_* = 2464.44	*F*(000) = 1214
Triclinic, *P*1	*D*_x_ = 2.380 Mg m^−^^3^
*a* = 9.6370 (4) Å	Cu *K*α radiation, λ = 1.54178 Å
*b* = 11.5888 (5) Å	Cell parameters from 9965 reflections
*c* = 16.2367 (6) Å	θ = 4.0–79.9°
α = 99.6716 (13)°	µ = 15.11 mm^−^^1^
β = 91.3031 (12)°	*T* = 150 K
γ = 105.3123 (12)°	Plate, blue
*V* = 1719.80 (12) Å^3^	0.20 × 0.20 × 0.08 mm

### Data collection

**Table:**

Bruker AXS D8 Quest CMOS diffractometer with PhotonII charge-integrating pixel array detector (CPAD)	7029 independent reflections
Radiation source: I-mu-S microsource X-ray tube	6786 reflections with *I* > 2σ(*I*)
Laterally graded multilayer (Goebel) mirror monochromator	*R*_int_ = 0.050
Detector resolution: 7.4074 pixels mm^-1^	θ_max_ = 80.3°, θ_min_ = 2.8°
ω and phi scans	*h* = −12→12
Absorption correction: multi-scan (SADABS; Krause *et al.*, 2015	*k* = −14→14
*T*_min_ = 0.454, *T*_max_ = 0.754	*l* = −19→15
16278 measured reflections	

### Refinement

**Table:**

Refinement on *F*^2^	Primary atom site location: structure-invariant direct methods
Least-squares matrix: full	Secondary atom site location: difference Fourier map
*R*[*F*^2^ > 2σ(*F*^2^)] = 0.041	Hydrogen site location: mixed
*wR*(*F*^2^) = 0.118	H atoms treated by a mixture of independent and constrained refinement
*S* = 1.10	*w* = 1/[σ^2^(*F*_o_^2^) + (0.0656*P*)^2^ + 1.8351*P*] where *P* = (*F*_o_^2^ + 2*F*_c_^2^)/3
7029 reflections	(Δ/σ)_max_ < 0.001
562 parameters	Δρ_max_ = 1.59 e Å^−^^3^
22 restraints	Δρ_min_ = −1.34 e Å^−^^3^

### Special details

**Table:**

Geometry. All esds (except the esd in the dihedral angle between two l.s. planes) are estimated using the full covariance matrix. The cell esds are taken into account individually in the estimation of esds in distances, angles and torsion angles; correlations between esds in cell parameters are only used when they are defined by crystal symmetry. An approximate (isotropic) treatment of cell esds is used for estimating esds involving l.s. planes.
Refinement. The structure is ismorphous with its Dy, Eu, Gd, Ho, Nd, Pr analogues (AVP85_10mz121, AVP355_10mz172, AVP621_09mz411 and AVP629_10mz194, AVP65_10mz125 and AVP651_10mz191, AVP70_10mz147, AVP75_10mz148 and AVP754_10mz650), and was solved by isomorphous replacement. The water molecule of O19 is disordered over two mutually exclusive positions across an inversion center and was refined as half occupied. The non-coordinated sulfate ion is located on an inversion center and the oxygen atoms are disordered over two sets of positions with half occupancy. Water H atom positions were refined and O-H distances were restrained to 0.84 (2) Angstrom, respectively. Some H···H distances were further restrained to 1.35 (2) Angstrom.

### Fractional atomic coordinates and isotropic or equivalent isotropic displacement parameters (Å^2^)

**Table:**

	*x*	*y*	*z*	*U*_iso_*/*U*_eq_	Occ. (<1)
C1	0.4433 (5)	0.2625 (4)	0.5042 (3)	0.0167 (8)	
C2	0.3610 (5)	0.2938 (4)	0.5788 (3)	0.0195 (9)	
H2C	0.320220	0.220082	0.602893	0.023*	
H2D	0.427084	0.354919	0.622292	0.023*	
C3	0.6783 (5)	0.0423 (4)	0.2493 (3)	0.0158 (8)	
C4	0.6931 (6)	−0.0360 (5)	0.3110 (3)	0.0235 (10)	
H4C	0.794720	−0.038433	0.316648	0.028*	
H4D	0.632740	−0.119964	0.290399	0.028*	
C5	0.7890 (5)	0.3483 (4)	0.0317 (3)	0.0175 (9)	
C6	0.8858 (5)	0.2707 (4)	−0.0019 (3)	0.0221 (10)	
H6C	0.874472	0.253424	−0.063895	0.027*	
H6D	0.987712	0.315424	0.015213	0.027*	
C7	0.5254 (5)	0.6985 (4)	0.1304 (3)	0.0159 (8)	
C8	0.6022 (5)	0.7507 (4)	0.0594 (3)	0.0179 (9)	
H8C	0.532673	0.736705	0.010561	0.022*	
H8D	0.643978	0.839491	0.076935	0.022*	
C9	0.2149 (5)	0.5808 (4)	0.3895 (3)	0.0163 (9)	
C10	0.1644 (5)	0.6898 (4)	0.3802 (3)	0.0222 (10)	
H10C	0.058903	0.664428	0.366493	0.027*	
H10D	0.184336	0.748022	0.434014	0.027*	
Cu1	0.57451 (7)	0.15475 (6)	0.38923 (4)	0.01792 (16)	
Cu2	0.71639 (7)	0.16393 (6)	0.12404 (4)	0.01884 (16)	
Cu3	0.68477 (7)	0.53540 (6)	0.08031 (4)	0.01553 (15)	
Cu4	0.34975 (7)	0.64627 (6)	0.24858 (4)	0.01548 (15)	
Cu5	0.28203 (7)	0.39948 (6)	0.44370 (4)	0.01615 (15)	
Tb1	0.48345 (2)	0.35681 (2)	0.24306 (2)	0.01322 (9)	
N1	0.4245 (4)	0.3141 (3)	0.4416 (2)	0.0171 (7)	
N2	0.2431 (4)	0.3430 (4)	0.5530 (2)	0.0195 (8)	
H2A	0.236548	0.406266	0.592889	0.023*	
H2B	0.157731	0.284301	0.547628	0.023*	
N3	0.6105 (4)	0.1242 (3)	0.2732 (2)	0.0178 (7)	
N4	0.6482 (5)	0.0108 (4)	0.3943 (3)	0.0215 (8)	
H4A	0.724750	0.031948	0.432837	0.026*	
H4B	0.577960	−0.048780	0.410697	0.026*	
N5	0.7027 (4)	0.3075 (3)	0.0865 (2)	0.0166 (7)	
N6	0.8492 (4)	0.1541 (3)	0.0304 (2)	0.0171 (7)	
H6A	0.931299	0.139490	0.049586	0.021*	
H6B	0.805401	0.091646	−0.011692	0.021*	
N7	0.5575 (4)	0.6039 (3)	0.1480 (2)	0.0172 (7)	
N8	0.7190 (4)	0.6918 (3)	0.0356 (2)	0.0159 (7)	
H8A	0.805912	0.742721	0.057077	0.019*	
H8B	0.720395	0.676114	−0.021109	0.019*	
N9	0.2929 (4)	0.5471 (3)	0.3302 (2)	0.0173 (7)	
N10	0.2378 (4)	0.7509 (4)	0.3133 (3)	0.0226 (8)	
H10A	0.298699	0.824200	0.336523	0.027*	
H10B	0.171092	0.763954	0.278010	0.027*	
O1	0.5000 (3)	0.2882 (3)	0.37163 (19)	0.0166 (6)	
O2	0.5244 (4)	0.1896 (3)	0.5060 (2)	0.0196 (6)	
O3	0.6029 (4)	0.1998 (3)	0.2163 (2)	0.0199 (7)	
O4	0.7336 (4)	0.0300 (3)	0.1769 (2)	0.0196 (7)	
O5	0.6158 (3)	0.3809 (3)	0.11866 (19)	0.0157 (6)	
O6	0.7979 (4)	0.4510 (3)	0.0074 (2)	0.0189 (6)	
O7	0.4861 (3)	0.5537 (3)	0.2123 (2)	0.0166 (6)	
O8	0.4330 (3)	0.7478 (3)	0.1689 (2)	0.0193 (6)	
O9	0.3463 (3)	0.4493 (3)	0.3393 (2)	0.0170 (6)	
O10	0.1827 (4)	0.5265 (3)	0.4519 (2)	0.0195 (7)	
O11	0.2853 (4)	0.1696 (3)	0.2229 (2)	0.0238 (7)	
O12	0.2734 (4)	0.3216 (3)	0.1464 (2)	0.0205 (7)	
O13	0.1448 (4)	0.1123 (3)	0.0876 (2)	0.0253 (7)	
O14	0.0575 (4)	0.2189 (4)	0.2058 (2)	0.0311 (8)	
O15	0.7222 (3)	0.4609 (3)	0.3006 (2)	0.0184 (6)	
H15A	0.767 (6)	0.430 (5)	0.331 (3)	0.028*	
H15B	0.782 (5)	0.495 (5)	0.269 (3)	0.028*	
O16	0.8851 (4)	0.5789 (3)	0.1920 (2)	0.0254 (7)	
H16A	0.960 (5)	0.557 (6)	0.184 (4)	0.038*	
H16B	0.917 (7)	0.653 (2)	0.213 (4)	0.038*	
O17	0.1408 (4)	0.5205 (3)	0.1525 (2)	0.0220 (7)	
H17A	0.163 (7)	0.457 (4)	0.156 (4)	0.033*	
H17B	0.155 (7)	0.528 (6)	0.1031 (18)	0.033*	
O18	0.0716 (4)	0.2521 (3)	0.3767 (2)	0.0241 (7)	
H18A	0.043 (7)	0.185 (3)	0.393 (4)	0.036*	
H18B	0.070 (7)	0.236 (6)	0.3246 (13)	0.036*	
O19	0.5200 (11)	0.0381 (11)	0.0275 (6)	0.047 (2)	0.5
H19A	0.472 (19)	−0.003 (15)	0.060 (9)	0.071*	0.5
H19B	0.55 (2)	0.02 (2)	−0.021 (6)	0.071*	0.5
O20	0.3102 (4)	0.0221 (3)	0.3519 (3)	0.0283 (8)	
H20A	0.236 (5)	0.022 (7)	0.377 (4)	0.043*	
H20B	0.291 (8)	0.063 (6)	0.318 (4)	0.043*	
O21	0.8274 (4)	0.3337 (4)	0.3966 (2)	0.0308 (8)	
H21A	0.909 (4)	0.320 (7)	0.392 (5)	0.046*	
H21B	0.841 (8)	0.380 (6)	0.443 (3)	0.046*	
O22	0.9749 (5)	0.8150 (4)	0.2711 (3)	0.0361 (9)	
H22A	0.972 (9)	0.855 (7)	0.319 (2)	0.054*	
H22B	0.980 (9)	0.857 (6)	0.234 (4)	0.054*	
O23	0.9394 (4)	0.9116 (3)	0.1345 (2)	0.0243 (7)	
H23A	0.876 (4)	0.947 (5)	0.150 (4)	0.036*	
H23B	1.011 (4)	0.968 (4)	0.126 (4)	0.036*	
O24	0.3431 (7)	0.9430 (4)	0.1264 (3)	0.0523 (14)	
H24A	0.328 (11)	0.998 (6)	0.163 (4)	0.079*	
H24B	0.368 (10)	0.893 (6)	0.152 (4)	0.079*	
O25	0.1618 (8)	0.0365 (9)	0.4920 (5)	0.0357 (19)	0.5
O26	−0.0300 (9)	−0.0158 (7)	0.5820 (5)	0.0339 (17)	0.5
O27	−0.0484 (9)	0.1032 (7)	0.4781 (5)	0.0333 (17)	0.5
O28	−0.0592 (9)	−0.1078 (7)	0.4360 (5)	0.0346 (17)	0.5
S1	0.18461 (12)	0.20240 (10)	0.16476 (7)	0.0195 (2)	
S2	0.000000	0.000000	0.500000	0.0199 (3)	

### Atomic displacement parameters (Å^2^)

**Table:**

	*U*^11^	*U*^22^	*U*^33^	*U*^12^	*U*^13^	*U*^23^
C1	0.0163 (19)	0.0162 (19)	0.019 (2)	0.0070 (16)	0.0042 (17)	0.0016 (17)
C2	0.024 (2)	0.025 (2)	0.016 (2)	0.0143 (18)	0.0049 (17)	0.0080 (18)
C3	0.0152 (19)	0.0150 (19)	0.019 (2)	0.0092 (16)	0.0030 (16)	0.0011 (17)
C4	0.034 (3)	0.024 (2)	0.019 (2)	0.020 (2)	0.0063 (19)	0.0025 (19)
C5	0.018 (2)	0.019 (2)	0.016 (2)	0.0074 (17)	0.0045 (17)	0.0023 (17)
C6	0.025 (2)	0.018 (2)	0.025 (2)	0.0068 (18)	0.0113 (19)	0.0018 (18)
C7	0.0150 (19)	0.0126 (19)	0.019 (2)	0.0023 (15)	0.0009 (16)	0.0027 (17)
C8	0.018 (2)	0.017 (2)	0.021 (2)	0.0078 (16)	0.0038 (17)	0.0055 (17)
C9	0.018 (2)	0.0165 (19)	0.017 (2)	0.0114 (16)	0.0011 (16)	−0.0027 (17)
C10	0.029 (2)	0.024 (2)	0.022 (2)	0.0194 (19)	0.0081 (19)	0.0066 (19)
Cu1	0.0252 (3)	0.0179 (3)	0.0165 (3)	0.0146 (3)	0.0054 (3)	0.0048 (3)
Cu2	0.0252 (3)	0.0152 (3)	0.0212 (4)	0.0122 (3)	0.0111 (3)	0.0051 (3)
Cu3	0.0188 (3)	0.0136 (3)	0.0167 (3)	0.0074 (2)	0.0067 (2)	0.0039 (2)
Cu4	0.0173 (3)	0.0138 (3)	0.0189 (3)	0.0090 (2)	0.0052 (2)	0.0045 (2)
Cu5	0.0188 (3)	0.0174 (3)	0.0169 (3)	0.0111 (3)	0.0069 (2)	0.0053 (3)
Tb1	0.01436 (14)	0.01192 (14)	0.01535 (15)	0.00683 (10)	0.00396 (10)	0.00235 (10)
N1	0.0233 (18)	0.0181 (17)	0.0138 (18)	0.0112 (15)	0.0065 (14)	0.0044 (14)
N2	0.0220 (19)	0.0194 (18)	0.020 (2)	0.0106 (15)	0.0073 (15)	0.0036 (15)
N3	0.0234 (19)	0.0150 (17)	0.0201 (19)	0.0111 (15)	0.0070 (15)	0.0071 (15)
N4	0.029 (2)	0.0223 (19)	0.020 (2)	0.0157 (16)	0.0087 (16)	0.0072 (16)
N5	0.0159 (17)	0.0179 (17)	0.0169 (18)	0.0087 (14)	0.0056 (14)	−0.0016 (15)
N6	0.0171 (17)	0.0183 (18)	0.0193 (19)	0.0092 (14)	0.0060 (14)	0.0053 (15)
N7	0.0192 (18)	0.0170 (17)	0.0174 (18)	0.0075 (14)	0.0036 (15)	0.0043 (15)
N8	0.0212 (18)	0.0104 (15)	0.0183 (18)	0.0060 (14)	0.0061 (14)	0.0057 (14)
N9	0.0202 (18)	0.0166 (17)	0.0185 (19)	0.0108 (14)	0.0029 (15)	0.0032 (15)
N10	0.0212 (19)	0.0169 (18)	0.034 (2)	0.0125 (15)	0.0102 (17)	0.0039 (17)
O1	0.0207 (15)	0.0205 (15)	0.0148 (15)	0.0141 (12)	0.0090 (12)	0.0057 (12)
O2	0.0277 (17)	0.0195 (15)	0.0172 (16)	0.0146 (13)	0.0065 (13)	0.0050 (13)
O3	0.0307 (17)	0.0187 (15)	0.0182 (16)	0.0155 (13)	0.0121 (13)	0.0095 (13)
O4	0.0251 (16)	0.0183 (15)	0.0197 (16)	0.0123 (13)	0.0084 (13)	0.0041 (13)
O5	0.0193 (14)	0.0119 (13)	0.0197 (16)	0.0103 (11)	0.0064 (12)	0.0031 (12)
O6	0.0271 (17)	0.0166 (14)	0.0172 (16)	0.0107 (13)	0.0104 (13)	0.0053 (12)
O7	0.0180 (14)	0.0154 (14)	0.0205 (16)	0.0086 (12)	0.0114 (12)	0.0067 (12)
O8	0.0201 (15)	0.0193 (15)	0.0238 (17)	0.0120 (12)	0.0072 (13)	0.0070 (13)
O9	0.0204 (15)	0.0178 (15)	0.0210 (16)	0.0168 (12)	0.0109 (12)	0.0065 (13)
O10	0.0280 (17)	0.0209 (15)	0.0172 (16)	0.0160 (13)	0.0094 (13)	0.0081 (13)
O11	0.0247 (17)	0.0219 (16)	0.0246 (18)	0.0061 (13)	−0.0008 (14)	0.0042 (14)
O12	0.0253 (16)	0.0174 (15)	0.0180 (16)	0.0028 (13)	0.0002 (13)	0.0058 (13)
O13	0.0280 (17)	0.0206 (16)	0.0241 (18)	0.0028 (14)	0.0013 (14)	0.0010 (14)
O14	0.0210 (17)	0.043 (2)	0.0267 (19)	0.0086 (16)	0.0047 (14)	−0.0019 (16)
O15	0.0178 (15)	0.0222 (16)	0.0167 (16)	0.0076 (12)	0.0016 (12)	0.0041 (13)
O16	0.0190 (16)	0.0268 (17)	0.0287 (19)	0.0047 (14)	0.0044 (14)	0.0029 (15)
O17	0.0281 (17)	0.0224 (16)	0.0183 (16)	0.0111 (14)	0.0040 (14)	0.0043 (14)
O18	0.0261 (17)	0.0227 (17)	0.0236 (18)	0.0066 (14)	0.0045 (14)	0.0040 (14)
O19	0.038 (5)	0.064 (6)	0.026 (4)	−0.007 (4)	−0.001 (4)	0.005 (4)
O20	0.0309 (19)	0.0200 (17)	0.035 (2)	0.0066 (15)	0.0100 (16)	0.0063 (15)
O21	0.0287 (19)	0.044 (2)	0.0241 (19)	0.0232 (17)	0.0003 (15)	−0.0036 (16)
O22	0.047 (2)	0.036 (2)	0.032 (2)	0.0236 (19)	−0.0020 (19)	0.0038 (17)
O23	0.0215 (16)	0.0173 (15)	0.035 (2)	0.0072 (13)	0.0068 (15)	0.0026 (14)
O24	0.090 (4)	0.039 (2)	0.037 (2)	0.043 (3)	−0.015 (2)	−0.008 (2)
O25	0.023 (4)	0.058 (5)	0.034 (4)	0.014 (4)	0.007 (3)	0.022 (4)
O26	0.042 (4)	0.034 (4)	0.025 (4)	0.011 (3)	0.007 (3)	0.002 (3)
O27	0.038 (4)	0.030 (4)	0.041 (5)	0.018 (3)	0.007 (3)	0.018 (3)
O28	0.044 (5)	0.026 (4)	0.033 (4)	0.009 (3)	0.004 (3)	0.003 (3)
S1	0.0186 (5)	0.0195 (5)	0.0200 (5)	0.0044 (4)	0.0019 (4)	0.0037 (4)
S2	0.0189 (7)	0.0180 (7)	0.0235 (8)	0.0071 (6)	−0.0005 (6)	0.0026 (6)

### Geometric parameters (Å, º)

**Table:**

C1—N1	1.294 (6)	Cu5—N2	2.003 (4)
C1—O2	1.298 (5)	Cu5—O18	2.379 (4)
C1—C2	1.509 (6)	Tb1—O9	2.370 (3)
C2—N2	1.480 (6)	Tb1—O1	2.372 (3)
C2—H2C	0.9900	Tb1—O15	2.383 (3)
C2—H2D	0.9900	Tb1—O3	2.386 (3)
C3—N3	1.301 (5)	Tb1—O7	2.411 (3)
C3—O4	1.304 (6)	Tb1—O5	2.430 (3)
C3—C4	1.488 (7)	Tb1—O12	2.436 (3)
C4—N4	1.488 (6)	Tb1—O11	2.451 (3)
C4—H4C	0.9900	Tb1—S1	3.0756 (11)
C4—H4D	0.9900	N1—O1	1.396 (5)
C5—N5	1.295 (6)	N2—H2A	0.9100
C5—O6	1.298 (6)	N2—H2B	0.9100
C5—C6	1.509 (6)	N3—O3	1.389 (5)
C6—N6	1.491 (6)	N4—H4A	0.9100
C6—H6C	0.9900	N4—H4B	0.9100
C6—H6D	0.9900	N5—O5	1.395 (4)
C7—N7	1.288 (6)	N6—H6A	0.9100
C7—O8	1.298 (5)	N6—H6B	0.9100
C7—C8	1.509 (6)	N7—O7	1.388 (5)
C8—N8	1.489 (5)	N8—H8A	0.9100
C8—H8C	0.9900	N8—H8B	0.9100
C8—H8D	0.9900	N9—O9	1.391 (5)
C9—O10	1.282 (6)	N10—H10A	0.9100
C9—N9	1.306 (6)	N10—H10B	0.9100
C9—C10	1.498 (6)	O11—S1	1.500 (4)
C10—N10	1.487 (7)	O12—S1	1.502 (3)
C10—H10C	0.9900	O13—S1	1.461 (3)
C10—H10D	0.9900	O14—S1	1.448 (4)
Cu1—N3	1.915 (4)	O15—H15A	0.84 (2)
Cu1—O1	1.928 (3)	O15—H15B	0.84 (2)
Cu1—O2	1.969 (3)	O16—H16A	0.84 (2)
Cu1—N4	1.991 (4)	O16—H16B	0.84 (2)
Cu1—O20	2.601 (4)	O17—H17A	0.83 (2)
Cu1—O21	2.736 (4)	O17—H17B	0.83 (2)
Cu2—N5	1.900 (4)	O18—H18A	0.84 (2)
Cu2—O3	1.928 (3)	O18—H18B	0.83 (2)
Cu2—O4	1.936 (3)	O19—H19A	0.84 (2)
Cu2—N6	2.018 (4)	O19—H19B	0.84 (2)
Cu2—O19	2.409 (10)	O20—H20A	0.83 (2)
Cu3—N7	1.904 (4)	O20—H20B	0.83 (2)
Cu3—O6	1.944 (3)	O21—H21A	0.84 (2)
Cu3—O5	1.949 (3)	O21—H21B	0.83 (2)
Cu3—N8	2.014 (4)	O22—H22A	0.84 (2)
Cu3—O16	2.508 (4)	O22—H22B	0.84 (2)
Cu4—N9	1.894 (4)	O23—H23A	0.84 (2)
Cu4—O8	1.940 (3)	O23—H23B	0.85 (2)
Cu4—O7	1.947 (3)	O24—H24A	0.84 (2)
Cu4—N10	2.012 (4)	O24—H24B	0.84 (2)
Cu4—O17	2.481 (4)	O25—S2	1.519 (7)
Cu5—N1	1.890 (4)	O26—S2	1.401 (8)
Cu5—O9	1.943 (3)	O27—S2	1.485 (7)
Cu5—O10	1.946 (3)	O28—S2	1.458 (8)
			
N1—C1—O2	125.3 (4)	O5—Tb1—O11	112.83 (11)
N1—C1—C2	114.2 (4)	O12—Tb1—O11	57.34 (11)
O2—C1—C2	120.5 (4)	O9—Tb1—S1	83.26 (8)
N2—C2—C1	110.0 (4)	O1—Tb1—S1	102.84 (8)
N2—C2—H2C	109.7	O15—Tb1—S1	174.96 (8)
C1—C2—H2C	109.7	O3—Tb1—S1	96.74 (9)
N2—C2—H2D	109.7	O7—Tb1—S1	101.43 (8)
C1—C2—H2D	109.7	O5—Tb1—S1	101.06 (8)
H2C—C2—H2D	108.2	O12—Tb1—S1	28.74 (8)
N3—C3—O4	123.0 (4)	O11—Tb1—S1	28.77 (8)
N3—C3—C4	115.9 (4)	C1—N1—O1	115.9 (3)
O4—C3—C4	121.1 (4)	C1—N1—Cu5	119.5 (3)
C3—C4—N4	111.1 (4)	O1—N1—Cu5	124.1 (3)
C3—C4—H4C	109.4	C2—N2—Cu5	109.8 (3)
N4—C4—H4C	109.4	C2—N2—H2A	109.7
C3—C4—H4D	109.4	Cu5—N2—H2A	109.7
N4—C4—H4D	109.4	C2—N2—H2B	109.7
H4C—C4—H4D	108.0	Cu5—N2—H2B	109.7
N5—C5—O6	123.7 (4)	H2A—N2—H2B	108.2
N5—C5—C6	116.0 (4)	C3—N3—O3	115.1 (4)
O6—C5—C6	120.3 (4)	C3—N3—Cu1	117.5 (3)
N6—C6—C5	110.5 (4)	O3—N3—Cu1	125.5 (3)
N6—C6—H6C	109.5	C4—N4—Cu1	110.6 (3)
C5—C6—H6C	109.5	C4—N4—H4A	109.5
N6—C6—H6D	109.5	Cu1—N4—H4A	109.5
C5—C6—H6D	109.5	C4—N4—H4B	109.5
H6C—C6—H6D	108.1	Cu1—N4—H4B	109.5
N7—C7—O8	124.1 (4)	H4A—N4—H4B	108.1
N7—C7—C8	115.5 (4)	C5—N5—O5	115.7 (4)
O8—C7—C8	120.4 (4)	C5—N5—Cu2	118.6 (3)
N8—C8—C7	109.8 (4)	O5—N5—Cu2	125.5 (3)
N8—C8—H8C	109.7	C6—N6—Cu2	109.6 (3)
C7—C8—H8C	109.7	C6—N6—H6A	109.7
N8—C8—H8D	109.7	Cu2—N6—H6A	109.7
C7—C8—H8D	109.7	C6—N6—H6B	109.7
H8C—C8—H8D	108.2	Cu2—N6—H6B	109.7
O10—C9—N9	123.8 (4)	H6A—N6—H6B	108.2
O10—C9—C10	121.2 (4)	C7—N7—O7	116.2 (4)
N9—C9—C10	115.0 (4)	C7—N7—Cu3	119.0 (3)
N10—C10—C9	111.3 (4)	O7—N7—Cu3	124.6 (3)
N10—C10—H10C	109.4	C8—N8—Cu3	109.7 (3)
C9—C10—H10C	109.4	C8—N8—H8A	109.7
N10—C10—H10D	109.4	Cu3—N8—H8A	109.7
C9—C10—H10D	109.4	C8—N8—H8B	109.7
H10C—C10—H10D	108.0	Cu3—N8—H8B	109.7
N3—Cu1—O1	90.36 (14)	H8A—N8—H8B	108.2
N3—Cu1—O2	175.68 (15)	C9—N9—O9	116.1 (4)
O1—Cu1—O2	86.12 (13)	C9—N9—Cu4	119.1 (3)
N3—Cu1—N4	83.85 (16)	O9—N9—Cu4	124.1 (3)
O1—Cu1—N4	173.91 (15)	C10—N10—Cu4	109.9 (3)
O2—Cu1—N4	99.57 (15)	C10—N10—H10A	109.7
N3—Cu1—O20	89.10 (15)	Cu4—N10—H10A	109.7
O1—Cu1—O20	85.07 (13)	C10—N10—H10B	109.7
O2—Cu1—O20	88.10 (14)	Cu4—N10—H10B	109.7
N4—Cu1—O20	92.89 (15)	H10A—N10—H10B	108.2
N3—Cu1—O21	82.42 (14)	N1—O1—Cu1	106.2 (2)
O1—Cu1—O21	80.09 (13)	N1—O1—Tb1	125.6 (2)
O2—Cu1—O21	99.41 (13)	Cu1—O1—Tb1	126.24 (14)
N4—Cu1—O21	100.98 (15)	C1—O2—Cu1	104.0 (3)
O20—Cu1—O21	162.82 (12)	N3—O3—Cu2	108.6 (2)
N5—Cu2—O3	89.51 (15)	N3—O3—Tb1	122.4 (2)
N5—Cu2—O4	172.53 (15)	Cu2—O3—Tb1	128.77 (15)
O3—Cu2—O4	84.79 (13)	C3—O4—Cu2	107.7 (3)
N5—Cu2—N6	83.61 (16)	N5—O5—Cu3	107.1 (2)
O3—Cu2—N6	171.24 (15)	N5—O5—Tb1	124.1 (2)
O4—Cu2—N6	101.58 (15)	Cu3—O5—Tb1	123.88 (13)
N5—Cu2—O19	92.3 (3)	C5—O6—Cu3	107.0 (3)
O3—Cu2—O19	97.4 (3)	N7—O7—Cu4	107.2 (2)
O4—Cu2—O19	93.2 (3)	N7—O7—Tb1	124.7 (2)
N6—Cu2—O19	88.3 (3)	Cu4—O7—Tb1	125.89 (14)
N7—Cu3—O6	174.11 (15)	C7—O8—Cu4	106.9 (3)
N7—Cu3—O5	91.24 (15)	N9—O9—Cu5	107.2 (2)
O6—Cu3—O5	84.79 (13)	N9—O9—Tb1	126.2 (2)
N7—Cu3—N8	82.67 (16)	Cu5—O9—Tb1	126.55 (14)
O6—Cu3—N8	100.63 (14)	C9—O10—Cu5	107.4 (3)
O5—Cu3—N8	169.87 (14)	S1—O11—Tb1	99.41 (17)
N7—Cu3—O16	96.61 (14)	S1—O12—Tb1	100.02 (16)
O6—Cu3—O16	87.37 (13)	Tb1—O15—H15A	121 (4)
O5—Cu3—O16	84.87 (13)	Tb1—O15—H15B	118 (4)
N8—Cu3—O16	103.81 (14)	H15A—O15—H15B	107 (6)
N9—Cu4—O8	172.67 (15)	Cu3—O16—H16A	122 (5)
N9—Cu4—O7	89.19 (15)	Cu3—O16—H16B	114 (5)
O8—Cu4—O7	85.34 (13)	H16A—O16—H16B	102 (7)
N9—Cu4—N10	83.73 (17)	Cu4—O17—H17A	92 (5)
O8—Cu4—N10	100.54 (16)	Cu4—O17—H17B	111 (5)
O7—Cu4—N10	165.82 (17)	H17A—O17—H17B	103 (6)
N9—Cu4—O17	90.56 (14)	Cu5—O18—H18A	120 (5)
O8—Cu4—O17	94.98 (13)	Cu5—O18—H18B	115 (5)
O7—Cu4—O17	97.53 (13)	H18A—O18—H18B	107 (7)
N10—Cu4—O17	94.81 (15)	Cu2—O19—H19A	99 (10)
N1—Cu5—O9	88.98 (14)	Cu2—O19—H19B	113 (10)
N1—Cu5—O10	163.89 (16)	H19A—O19—H19B	135 (10)
O9—Cu5—O10	85.41 (13)	Cu1—O20—H20A	131 (5)
N1—Cu5—N2	83.21 (16)	Cu1—O20—H20B	95 (5)
O9—Cu5—N2	171.78 (14)	H20A—O20—H20B	93 (7)
O10—Cu5—N2	101.44 (14)	Cu1—O21—H21A	124 (5)
N1—Cu5—O18	104.51 (15)	Cu1—O21—H21B	110 (5)
O9—Cu5—O18	93.14 (13)	H21A—O21—H21B	101 (7)
O10—Cu5—O18	90.88 (14)	H22A—O22—H22B	113 (8)
N2—Cu5—O18	91.31 (15)	H23A—O23—H23B	105 (3)
O9—Tb1—O1	71.67 (10)	H24A—O24—H24B	108 (3)
O9—Tb1—O15	100.80 (11)	O14—S1—O13	110.9 (2)
O1—Tb1—O15	75.87 (11)	O14—S1—O11	111.0 (2)
O9—Tb1—O3	144.63 (11)	O13—S1—O11	111.6 (2)
O1—Tb1—O3	73.91 (10)	O14—S1—O12	110.1 (2)
O15—Tb1—O3	78.22 (11)	O13—S1—O12	110.3 (2)
O9—Tb1—O7	70.65 (10)	O11—S1—O12	102.68 (19)
O1—Tb1—O7	131.81 (10)	O14—S1—Tb1	118.92 (16)
O15—Tb1—O7	82.82 (11)	O13—S1—Tb1	130.15 (15)
O3—Tb1—O7	142.47 (10)	O11—S1—Tb1	51.82 (13)
O9—Tb1—O5	143.39 (10)	O12—S1—Tb1	51.24 (13)
O1—Tb1—O5	139.79 (10)	O26^i^—S2—O26	180.0
O15—Tb1—O5	77.50 (11)	O26^i^—S2—O28^i^	114.8 (5)
O3—Tb1—O5	71.55 (10)	O26—S2—O28^i^	65.2 (5)
O7—Tb1—O5	72.88 (10)	O26^i^—S2—O28	65.2 (5)
O9—Tb1—O12	83.74 (11)	O26—S2—O28	114.8 (5)
O1—Tb1—O12	129.16 (11)	O28^i^—S2—O28	180.0 (5)
O15—Tb1—O12	153.99 (11)	O26^i^—S2—O27	68.7 (5)
O3—Tb1—O12	112.70 (11)	O26—S2—O27	111.3 (5)
O7—Tb1—O12	74.50 (11)	O28^i^—S2—O27	70.8 (5)
O5—Tb1—O12	83.70 (11)	O28—S2—O27	109.2 (5)
O9—Tb1—O11	88.43 (11)	O26^i^—S2—O25	69.6 (5)
O1—Tb1—O11	77.71 (11)	O26—S2—O25	110.4 (5)
O15—Tb1—O11	147.49 (12)	O28^i^—S2—O25	73.6 (5)
O3—Tb1—O11	76.51 (12)	O28—S2—O25	106.4 (5)
O7—Tb1—O11	129.39 (11)	O27—S2—O25	104.2 (5)
			
N1—C1—C2—N2	18.5 (6)	O10—C9—N9—Cu4	−173.1 (3)
O2—C1—C2—N2	−161.5 (4)	C10—C9—N9—Cu4	6.7 (5)
N3—C3—C4—N4	−9.9 (6)	O7—Cu4—N9—C9	167.0 (4)
O4—C3—C4—N4	168.5 (4)	N10—Cu4—N9—C9	−0.7 (4)
N5—C5—C6—N6	5.8 (6)	O17—Cu4—N9—C9	−95.5 (3)
O6—C5—C6—N6	−176.2 (4)	O7—Cu4—N9—O9	−2.8 (3)
N7—C7—C8—N8	−10.1 (5)	N10—Cu4—N9—O9	−170.5 (3)
O8—C7—C8—N8	170.5 (4)	O17—Cu4—N9—O9	94.8 (3)
O10—C9—C10—N10	168.9 (4)	C9—C10—N10—Cu4	9.8 (5)
N9—C9—C10—N10	−10.9 (6)	C1—N1—O1—Cu1	11.7 (4)
O2—C1—N1—O1	−0.6 (6)	Cu5—N1—O1—Cu1	−159.6 (2)
C2—C1—N1—O1	179.4 (4)	C1—N1—O1—Tb1	176.5 (3)
O2—C1—N1—Cu5	171.2 (3)	Cu5—N1—O1—Tb1	5.2 (5)
C2—C1—N1—Cu5	−8.8 (5)	N1—C1—O2—Cu1	−10.6 (5)
O9—Cu5—N1—C1	175.3 (4)	C2—C1—O2—Cu1	169.4 (3)
O10—Cu5—N1—C1	105.8 (6)	C3—N3—O3—Cu2	−5.7 (4)
N2—Cu5—N1—C1	−2.1 (4)	Cu1—N3—O3—Cu2	158.1 (2)
O18—Cu5—N1—C1	−91.7 (4)	C3—N3—O3—Tb1	179.5 (3)
O9—Cu5—N1—O1	−13.6 (3)	Cu1—N3—O3—Tb1	−16.7 (5)
O10—Cu5—N1—O1	−83.1 (6)	N3—C3—O4—Cu2	7.1 (5)
N2—Cu5—N1—O1	169.0 (3)	C4—C3—O4—Cu2	−171.2 (4)
O18—Cu5—N1—O1	79.4 (3)	C5—N5—O5—Cu3	−9.7 (4)
C1—C2—N2—Cu5	−19.1 (5)	Cu2—N5—O5—Cu3	164.1 (2)
O4—C3—N3—O3	−1.0 (6)	C5—N5—O5—Tb1	−165.6 (3)
C4—C3—N3—O3	177.4 (4)	Cu2—N5—O5—Tb1	8.2 (4)
O4—C3—N3—Cu1	−166.2 (3)	N5—C5—O6—Cu3	8.8 (5)
C4—C3—N3—Cu1	12.2 (5)	C6—C5—O6—Cu3	−169.1 (3)
C3—C4—N4—Cu1	3.3 (5)	C7—N7—O7—Cu4	3.2 (4)
O6—C5—N5—O5	0.7 (6)	Cu3—N7—O7—Cu4	−171.6 (2)
C6—C5—N5—O5	178.6 (4)	C7—N7—O7—Tb1	167.2 (3)
O6—C5—N5—Cu2	−173.6 (3)	Cu3—N7—O7—Tb1	−7.7 (5)
C6—C5—N5—Cu2	4.4 (5)	N7—C7—O8—Cu4	−3.7 (5)
O3—Cu2—N5—C5	165.2 (4)	C8—C7—O8—Cu4	175.7 (3)
N6—Cu2—N5—C5	−9.4 (3)	C9—N9—O9—Cu5	−0.7 (4)
O19—Cu2—N5—C5	−97.4 (4)	Cu4—N9—O9—Cu5	169.3 (2)
O3—Cu2—N5—O5	−8.4 (3)	C9—N9—O9—Tb1	177.4 (3)
N6—Cu2—N5—O5	177.0 (3)	Cu4—N9—O9—Tb1	−12.5 (5)
O19—Cu2—N5—O5	88.9 (4)	N9—C9—O10—Cu5	4.2 (5)
C5—C6—N6—Cu2	−12.1 (5)	C10—C9—O10—Cu5	−175.6 (4)
O8—C7—N7—O7	0.3 (6)	Tb1—O11—S1—O14	−110.9 (2)
C8—C7—N7—O7	−179.1 (4)	Tb1—O11—S1—O13	124.81 (18)
O8—C7—N7—Cu3	175.5 (3)	Tb1—O11—S1—O12	6.7 (2)
C8—C7—N7—Cu3	−3.9 (5)	Tb1—O12—S1—O14	111.5 (2)
C7—C8—N8—Cu3	18.0 (4)	Tb1—O12—S1—O13	−125.78 (18)
O10—C9—N9—O9	−2.5 (6)	Tb1—O12—S1—O11	−6.7 (2)
C10—C9—N9—O9	177.3 (4)		

Symmetry code: (i) −*x*, −*y*, −*z*+1.

### Hydrogen-bond geometry (Å, º)

**Table:**

*D*—H···*A*	*D*—H	H···*A*	*D*···*A*	*D*—H···*A*
O24—H24*B*···O8	0.84 (2)	2.01 (3)	2.807 (5)	159 (7)
O24—H24*A*···O11^ii^	0.84 (2)	2.21 (3)	3.015 (5)	162 (7)
O23—H23*B*···O13^iii^	0.85 (2)	2.02 (3)	2.853 (5)	166 (6)
O23—H23*A*···O4^ii^	0.84 (2)	1.89 (2)	2.734 (5)	176 (7)
O22—H22*B*···O23	0.84 (2)	1.89 (3)	2.701 (6)	162 (8)
O22—H22*A*···O26^iv^	0.84 (2)	2.18 (4)	2.968 (9)	155 (8)
O22—H22*A*···O28^iii^	0.84 (2)	1.92 (3)	2.733 (9)	161 (8)
O21—H21*B*···O10^iv^	0.83 (2)	1.91 (3)	2.728 (5)	165 (8)
O21—H21*A*···O18^v^	0.84 (2)	1.94 (3)	2.765 (5)	167 (7)
O20—H20*B*···O11	0.83 (2)	2.14 (3)	2.960 (5)	168 (7)
O20—H20*A*···O26^i^	0.83 (2)	2.09 (3)	2.916 (9)	170 (7)
O20—H20*A*···O25	0.83 (2)	2.02 (5)	2.719 (9)	142 (7)
O19—H19*B*···O24^vi^	0.84 (2)	2.07 (9)	2.866 (11)	157 (22)
O19—H19*A*···O24^vii^	0.84 (2)	1.72 (7)	2.535 (12)	162 (21)
O18—H18*B*···O14	0.83 (2)	1.90 (2)	2.732 (5)	173 (7)
O18—H18*A*···O26^i^	0.84 (2)	2.04 (3)	2.857 (9)	163 (7)
O18—H18*A*···O27	0.84 (2)	1.91 (4)	2.648 (9)	146 (6)
O17—H17*B*···O6^vi^	0.83 (2)	1.90 (2)	2.730 (5)	176 (7)
O17—H17*A*···O12	0.83 (2)	2.10 (3)	2.905 (5)	163 (6)
O16—H16*B*···O22	0.84 (2)	1.89 (2)	2.721 (6)	173 (7)
O16—H16*A*···O17^v^	0.84 (2)	1.95 (2)	2.784 (5)	172 (7)
O15—H15*B*···O16	0.84 (2)	1.86 (2)	2.692 (5)	170 (6)
O15—H15*A*···O21	0.84 (2)	1.85 (3)	2.668 (5)	166 (6)
N10—H10*B*···O22^viii^	0.91	2.13	2.920 (6)	145
N10—H10*A*···O20^ii^	0.91	2.24	2.987 (5)	139
N8—H8*B*···O12^vi^	0.91	2.04	2.937 (5)	168
N8—H8*A*···O23	0.91	2.20	3.031 (5)	152
N6—H6*B*···O13^ix^	0.91	2.64	3.363 (5)	137
N6—H6*B*···O24^vi^	0.91	2.24	2.984 (6)	139
N6—H6*A*···O13^v^	0.91	2.25	3.158 (5)	175
N4—H4*B*···O2^x^	0.91	2.33	3.182 (5)	156
N4—H4*A*···O27^v^	0.91	2.18	3.037 (9)	156
N4—H4*A*···O25^x^	0.91	2.01	2.789 (9)	143
N2—H2*B*···O27	0.91	2.55	3.418 (9)	159
N2—H2*B*···O28^i^	0.91	2.08	2.868 (9)	144
N2—H2*A*···O15^iv^	0.91	2.07	2.946 (5)	162

Symmetry codes: (i) −*x*, −*y*, −*z*+1; (ii) *x*, *y*+1, *z*; (iii) *x*+1, *y*+1, *z*; (iv) −*x*+1, −*y*+1, −*z*+1; (v) *x*+1, *y*, *z*; (vi) −*x*+1, −*y*+1, −*z*; (vii) *x*, *y*−1, *z*; (viii) *x*−1, *y*, *z*; (ix) −*x*+1, −*y*, −*z*; (x) −*x*+1, −*y*, −*z*+1.
